# Journal of *Educational Evaluation for Health Professions* will be accepted for inclusion in Scopus

**DOI:** 10.3352/jeehp.2019.16.2

**Published:** 2019-01-07

**Authors:** Sun Huh

**Affiliations:** Department of Parasitology and Institute of Medical Education, Hallym University College of Medicine, Chuncheon, Korea; The Catholic University of Korea, Korea

On the morning of January 6, 2019, I received an email from the Scopus Title Evaluation Team stating that “The title mentioned above (*Journal of Educational Evaluation for Health Professions*, JEEHP) has been evaluated for inclusion in Scopus by the Content Selection & Advisory Board (CSAB). The review of this title is now complete and the CSAB has advised that the title will be accepted for inclusion in Scopus.”

Since 2016, JEEHP has already been searchable through Scopus as a MEDLINE-sourced journal [1]. However, being accepted by the CSAB yields the further advantage that references of the journal will be added to the Scopus database, making it easier to trace more expanded journal metrics. In contrast, for MEDLINE-sourced Scopus titles, only citation information drawn from the MEDLINE database is added to Scopus.

I am happy to share this announcement with the readers, authors, and reviewers of JEEHP. Without their help, it would have been impossible to be accepted by the Scopus CSAB. It may be worthwhile to present the chronological details of the journal’s application process to Scopus, since this information will help editors of society or non-profit organization journals to understand the process.

December 2004: The inaugural issue was published with 9 articles.

December 2006: The journal changed its official title to English from Korean (*Pogŏn ŭiryo kyoyuk pʻyŏngka*; ISSN 1738-1339), adopted in an English-only policy, and became an online-only journal without publication frequency [2]. It published 1 editorial, 1 review, 3 research articles, and 1 book review in 2006.

February 19, 2009: The full text of the journal began to be deposited to PubMed Central, and it became searchable through PubMed.

December 2010: The journal published 2 editorials, 1 technical report, 1 educational/faculty development material, and 2 brief reports in 2010.

September 28, 2011: The first application to Scopus was submitted.

February 21, 2013: The title was rejected. The comments were as follows: “Citedness is below expectations. There is no international diversity among authors. Only one issue is published per year. Also, author instructions need to be described more in detail.”

March 3, 2016: The journal began to be indexed in MEDLINE, making the citation data searchable in Scopus as a MEDLINE-sourced title starting with all issues of 2016 [1].

May 10, 2015: The second application to Scopus was submitted.

May 17, 2016: The outcome from the Scopus CSAB was rejection. The reason for making this decision was: “The citedness is below expectations and there is no international diversity among authors. Publication schedule per year is one issue only. Also, author instructions need to be described more in detail. The journal covers poor quality of presented research and a too broad area is covered in the journal” [3].

February 28, 2018: The third application to Scopus was submitted.

January 5, 2019: The title was accepted, with the following reviewer comments: “It would be preferable for market and geographic clarity if the journal adopted the title of ‘The Korean Journal of ...’ This journal will be relatively weak in the international rankings, and the editors still have a lot of work to improve the quality and quantity of content.”

Seven years and 4 months passed between first applying and being accepted by the Scopus CSAB. Their 3 rounds of comments for improvement can be summarized as follows:

(1) Title of ‘The Korean Journal of ...’ is preferable.

(2) Too broad of an area is covered in the journal.

(3) The author instructions need to be described more in detail

(4) Only one issue is published per year. The number of articles is small.

(5) There is no international diversity among authors.

(6) Citedness is below expectations. It is weak in the international rankings.

(7) The journal covers poor quality of presented research.

I and the editorial team have done our best to overcome the above weaknesses. Turning to the above-listed recommendations, it would be difficult to change the title to ‘The Korean Journal of ...’ because JEEHP has already become widely known throughout the world under its present title. Furthermore, authors from Korea accounted for only 21.1% of the articles and reviews published from 2015 to 2018. The top-ranking country of authors is the United States (31.3%). The 147 articles and reviews published during this period were from 48 countries (Fig. 1). Therefore, there is no clear evidence-based reason to change the journal title to ‘The Korean Journal of ...’ at present.

Each journal should have its own specific scope, and the scope of JEEHP is limited to educational evaluations in the field of medical health education. There is no other title in Scopus that has the same scope. One title in Scopus, *Evaluation and the Health Professions*, deals with evaluations; however, in addition to educational evaluations, its scope extends to health programs, teaching and training services, and products. However more precise description of the scope was added such as adoption of measurement theory to medical health education.

The author instructions were revised to contain a full description, including general information, research and publication ethics, manuscript preparation, manuscript submission, article processing charge, the peer review and publication process, principles of transparency and best practice of scholarly publishing, copyright information, open access policy, open access license agreement, and contact address, and are available at the journal homepage (https://jeehp.org).

As an online-only journal, JEEHP has no publication frequency, and therefore no issue numbers. This is a common feature of online-only journals throughout the world. Nonetheless, the number of publications remains insufficient. One hundred publications annually might be a standard for medical journals, but our narrow scope makes it difficult for us to recruit more articles while retaining high quality requirements. Furthermore, few articles published anywhere in the world deal with licensing examinations. To increase the number of publications is an urgent task.

The breadth of countries of authors provides evidence of the internationality of a journal. The number of authors’ countries has increased from 2 countries in 2006 to 24 in 2015, 34 in 2016, 18 in 2017, and 11 in 2018. Room remains for improvement, but based on the number of publications, this variety of countries provides good evidence of the internationality of JEEHP’s authorship.

Citedness reflects the international ranking and quality of a journal. There was a total of only 1 citation in 2008, but this metric soared to 31 in 2015 according to the Web of Science [4]. Cites per document (2 years) and total cites based on the Scopus database are presented in Figs. 2 and 3. In this calculation, self-citations were excluded. The most recent cites per document per 2 years (1.04) corresponds to a 2017 Scimago Journal ranking (SJR) of 1,435th out of 2,863 (49.9%) journals in the category of miscellaneous medicine, and a 2018 SJR ranking of 349th out of 1,262 (72.3%) in the category of education. When the analysis is limited to journals in the category of education in the Asia region, it ranks third out of 37 journals (91.1%). Nonetheless, I believe that this value of 2-year cites per document is still insufficient for JEEHP to be an internationally-branded journal. The number of total cites has increased year by year, from 2 in 2010 to 182 in 2018 (Fig. 3). However, a challenge in receiving more frequent citations from Scopus journals is that JEEHP has published several articles on country- or region-level licensing examinations, but relatively few articles are published throughout the world on licensing examinations for health professions. This phenomenon may be due to limitations in data access. If publications on licensing examinations become more frequent in the literature, the citation frequency will increase more rapidly.

As for the quality of the articles themselves, it is difficult for me to respond to this comment. Usually, quality refers to scientific soundness, creativeness, and applicability to the educational field. Creativeness is particularly difficult to assess because perceptions of creativeness vary according the readers’ region and field. However, the applicability of the articles published in JEEHP to the field can be affirmed with confidence, because most articles have analyzed classes in real-world educational settings or licensing examinations, not theoretical assumptions. Scientific soundness derives from reproducibility of the data analysis, the use of concrete and appropriate statistical analyses, the adoption of reporting guidelines, and logical interpretations of the data. As editor-in-chief, I adopted an open data policy to ensure reproducibility, and I have always tried my best to help authors present their work more lucidly, simply, and scientifically. If there are any poor-quality articles, it is due to the editor’s level of editing and understanding.

I appreciate the CSAB members whose comments have proven invaluable for the journal. Those comments spurred me to develop the journal according the Scopus journal selection criteria. Although JEEHP was accepted by the Scopus CSAB, it continues to face urgent tasks. First, the number of submissions should be increased, in order to increase the number of publications. Second, more articles on the application of measurement theory to medical health education should be recruited.

Another remarkable aspect of the performance of this journal, besides its content, is that it has been a pioneer in journal publishing in Korea. It has been published online-only since 2006, with PubMed Central XML production. It was the first scholarly journal in Korea to adopt PubMed Central XML in online journal publishing. Doing so was possible by coding XML tags for the full text of articles in July 2006. PubMed Central XML evolved into JATS (journal article tag suite) XML [5]. Second, it implemented audio or video recordings of abstracts by the authors in their mother tongue or in English on May 27, 2013 [6]. Third, ORCID (Open Researcher and Contributor ID) information began to be added for all authors on June 31, 2013 [7]. Fourth, the Crossmark (check for updates) and Funder Registry services by Crossref were introduced on August 13, 2013 [8]. Fifth, the Crossref text and data mining service was adopted on October 15, 2014. Sixth, an open data policy was adopted on March 24, 2016. Seventh, a clinical data sharing policy was introduced, according to ‘data sharing statements for clinical trials: a requirement of the International Committee of Medical Journal Editors,’ on January 8, 2018. Eighth, the third version of the ‘principles of transparency and best practice in scholarly publishing’ was implemented on July 15, 2018 [9]. The above 8 accomplishments are pioneering achievements in the history of scholarly journal publishing in Korea. It is hard for a local, small journal editor to compete with other international journals published by large commercial companies. To keep pace with them, I have tried my best to adopt new standards of scholarly journal publishing after discussions with publishers and editors at editors’ or publishers’ conferences [10].

I am proud to have been invited to be an editor of this journal, published by a public organization, the Korea Health Professions Licensing Examination Institute, where my senior physician and researcher colleagues have worked and carried out leadership roles with great devotion. I and the editorial team will strive to make the journal more readable and to be well-appreciated by medical and health instructors throughout the world.

## Figures and Tables

**Fig. 1.**
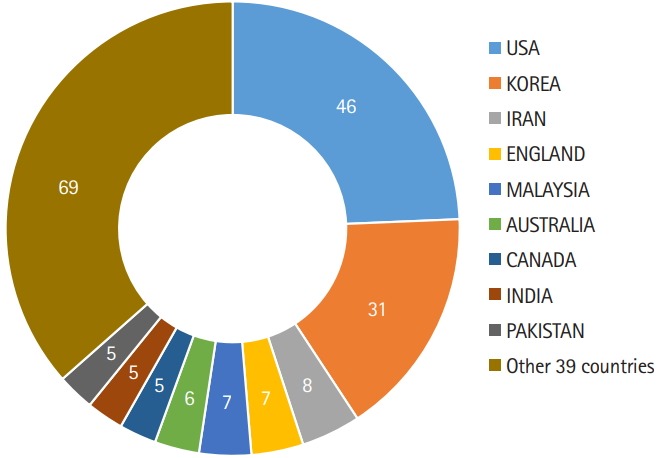
Countries of the authors of review and research articles published in *Journal of Educational Evaluation for Health Professions* from 2015 to 2018.

**Fig. 2.**
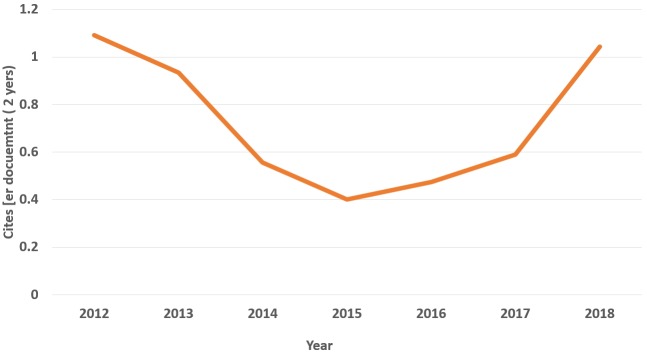
Cites per document (2 years) of articles published in *Journal of Educational Evaluation for Health Professions* from 2012 to 2018 based on the Scopus database.

**Fig. 3.**
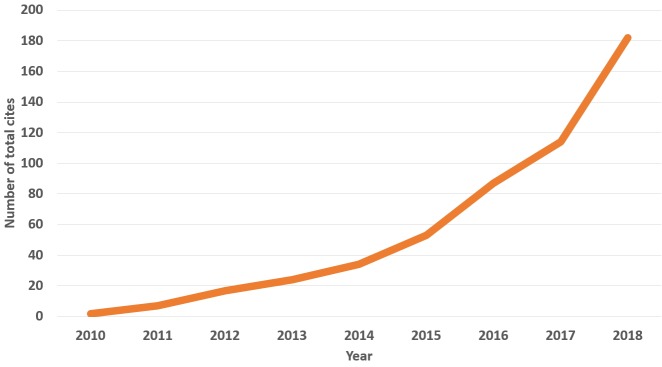
Total cites of *Journal of Educational Evaluation for Health Professions* from 2010 to 2018 based on the Scopus database.
